# Proteomic Analysis Reveals Sex-Specific Protein Degradation Targets in the Amygdala During Fear Memory Formation

**DOI:** 10.3389/fnmol.2021.716284

**Published:** 2021-09-29

**Authors:** Kayla Farrell, Madeline Musaus, Shaghayegh Navabpour, Kiley Martin, W. Keith Ray, Richard F. Helm, Timothy J. Jarome

**Affiliations:** ^1^Department of Animal and Poultry Science, Virginia Polytechnic Institute and State University, Blacksburg, VA, United States; ^2^School of Neuroscience, Virginia Polytechnic Institute and State University, Blacksburg, VA, United States; ^3^Department of Translational Biology, Medicine and Health, Virginia Polytechnic Institute and State University, Roanoke, VA, United States; ^4^Department of Biochemistry, Virginia Polytechnic Institute and State University, Blacksburg, VA, United States

**Keywords:** ubiquitin, proteasome, amygdala, sex difference, memory, consolidation

## Abstract

Ubiquitin-proteasome mediated protein degradation has been widely implicated in fear memory formation in the amygdala. However, to date, the protein targets of the proteasome remain largely unknown, limiting our understanding of the functional significance for protein degradation in fear memory formation. Additionally, whether similar proteins are targeted by the proteasome between sexes has yet to be explored. Here, we combined a degradation-specific K48 Tandem Ubiquitin Binding Entity (TUBE) with liquid chromatography mass spectrometry (LC/MS) to identify the target substrates of the protein degradation process in the amygdala of male and female rats following contextual fear conditioning. We found that males (43) and females (77) differed in the total number of proteins that had significant changes in K48 polyubiquitin targeting in the amygdala following fear conditioning. Many of the identified proteins (106) had significantly reduced levels in the K48-purified samples 1 h after fear conditioning, suggesting active degradation of the substrate due to learning. Interestingly, only 3 proteins overlapped between sexes, suggesting that targets of the protein degradation process may be sex-specific. In females, many proteins with altered abundance in the K48-purified samples were involved in vesicle transport or are associated with microtubules. Conversely, in males, proteins involved in the cytoskeleton, ATP synthesis and cell signaling were found to have significantly altered abundance. Only 1 protein had an opposite directional change in abundance between sexes, LENG1, which was significantly enhanced in males while lower in females. This suggests a more rapid degradation of this protein in females during fear memory formation. Interestingly, GFAP, a critical component of astrocyte structure, was a target of K48 polyubiquitination in both males and females, indicating that protein degradation is likely occurring in astrocytes following fear conditioning. Western blot assays revealed reduced levels of these target substrates following fear conditioning in both sexes, confirming that the K48 polyubiquitin was targeting these proteins for degradation. Collectively, this study provides strong evidence that sex differences exist in the protein targets of the degradation process in the amygdala following fear conditioning and critical information regarding how ubiquitin-proteasome mediated protein degradation may contribute to fear memory formation in the brain.

## Introduction

Post-traumatic stress disorder (PTSD) is a chronic anxiety disorder that affects nearly 5% of the world population. Despite this, treatment options are limited and none currently exist that can consistently reverse the symptoms of this disorder in all patients (Kessler et al., 2017, 2018). Importantly, females are more likely than males to develop PTSD despite not reporting experiencing more traumatic events (Breslau et al., 1997; Olff, 2017), though the mechanisms controlling this sex-dependent predisposition remain equivocal. A better understanding of the mechanisms controlling these sex differences in PTSD is important for developing therapeutic strategies that are effective in both male and female patients.

The ubiquitin-proteasome system (UPS) is a highly conserved pathway that controls the majority of protein turnover in cells (Bedford et al., 2010; Hegde, 2010; Reis et al., 2013; Hegde et al., 2014; Livneh et al., 2016; Collins and Goldberg, 2017) and dysregulation of this system has been widely implicated in a variety of neurological, neurodegenerative and psychiatric disorders (Dennissen et al., 2012; Zheng et al., 2016). This pathway is tightly regulated as proteins must first be “tagged” with the small regulatory protein ubiquitin *via* various E1, E2, and E3 ligases (Hershko and Ciechanover, 1998; Glickman and Ciechanover, 2002). Proteins may be monoubiquitinated or obtain multiple ubiquitin modifications, called polyubiquitination, the latter of which usually leads to degradation, though not all polyubiquitinated proteins are degraded. Ubiquitin can form different chains due to the presence of multiple lysine (K) residues on it which act as linkage sites (Dikic et al., 2009; Akutsu et al., 2016; Grice and Nathan, 2016). Lysine 48 (K48) polyubiquitin chains are highly abundant in cells and act as the canonical degradation signal (Newton et al., 2008).

Numerous studies conducted during the last two decades provide strong evidence to support a critical role of the UPS in the formation of fear memories that may underlie PTSD. It is well established that both degradation-specific (K48) polyubiquitin chains and proteasome activity are enhanced following fear learning in various brain regions, including the amygdala, hippocampus and prefrontal cortex (Lopez-Salon et al., 2001; Jarome et al., 2011; Rosenberg et al., 2016; Cullen et al., 2017; Orsi et al., 2019; Dulka et al., 2020). Additionally, the infusion of proteasome inhibitors into these brain regions leads to impaired memory for several different aversive and non-aversive behavioral paradigms (Rodriguez-Ortiz et al., 2011; Reis et al., 2013; Figueiredo et al., 2015; Furini et al., 2015; Rosenberg et al., 2016; Cullen et al., 2017). However, the majority of studies have been conducted solely in male rodents, though recent work from our group shows that a sex effect exists in the role of the UPS in fear memory formation (Devulapalli et al., 2021). This study demonstrated that while males, but not females, had increased UPS-mediated protein degradation during fear memory formation, genetic manipulations of ubiquitin and the proteasome impaired memory in both sexes, suggesting that despite these differences, protein degradation *via* the UPS is in fact necessary for fear memory formation in males and females. These findings indicate that males and females may differ in the functional role of ubiquitin-proteasome activity during fear memory formation, though this has never been tested. Furthermore, males and females differ in the age-related engagement of the protein degradation process following fear memory retrieval (Dulka et al., 2021), further emphasizing the importance of sex in studying UPS function during fear memory formation and disease-associated memory loss.

Although evidence supports an important role of protein degradation in fear memory formation, the proteins being targeted by the proteasome remain largely unknown. To date, only 4 proteins have been identified as putative targets of the proteasome during any stage of memory storage, including the synaptic scaffolds SHANK and GKAP, the transcriptional repressor IκB, and the RNA-induced silencing complex (RISC) factor MOV10 (Lopez-Salon et al., 2001; Lee et al., 2008; Jarome et al., 2011). All of these proteins were identified *via* candidate-driven approaches that rely on protein precipitation methods followed by immunoblotting, which can be non-specific and lack the sensitivity of unbiased proteomic approaches such as liquid chromatography mass spectrometry (LC/MS). Additionally, the primary method used in these studies, GST-S5a pull downs (Kamitani et al., 1997), cannot distinguish between the different types of polyubiquitin modifications, which have diverse functions in cells. The recent development of Tandem Ubiquitin Binding Entities (TUBEs) allow direct capture of specific forms of polyubiquitinated proteins in cells (Hjerpe et al., 2009; Mattern et al., 2019), which when combined with LC/MS can provide an unbiased, whole proteome analysis of linkage-specific protein polyubiquitination in cell lysates (Lopitz-Otsoa et al., 2012). However, to the best of our knowlegde, no unbiased examination of degradation-specific K48 polyubiquitinated proteins has ever been completed in the brain or during fear memory formation.

In order to better understand the functional role of the UPS in fear memory formation, the proteins being targeted for degradation (K48) following learning must be elucidated. Additionally, due to previous evidence suggesting differences in UPS levels between sexes, it is imperative that protein targets be identified in both sexes to evaluate whether the UPS acts in similar or different pathways to support fear memory formation. To address this gap in knowledge, we identified protein targets of K48 polyubiquitination in the basolateral amygdala of male and female rats during fear memory formation using K48-specific TUBEs in combination with LC/MS. We identified numerous protein targets of K48 polyubiquitination following learning, with very little overlap between sexes, implicating a variety of different cellular processes as being regulated by sex- and degradation-specific K48 polyubiquitination during fear memory formation.

## Methods and Materials

### Subjects

All experiments used 8–9 week old male or female Sprague Dawley rats obtained from Envigo (Frederick, MA). Animals were housed two per cage with free access to water and rat chow. The colony was maintained under a 12:12-h light/dark cycle. All experiments took place during the light portion of the cycle. All procedures were approved by the Virginia Polytechnic Institute and State University Institutional Animal Care and Use Committee (protocol #18-019) and conducted with the ethical guidelines of the National Institutes of Health.

### Apparatus

The 2 identical Habitest chambers used for contextual fear conditioning have been previously described in detail (Orsi et al., 2019). Habitest chamber consisted of a steel test cage with front and back Plexiglas walls and a grid shock floor above a plastic drop pan. The right wall of the chamber consisted of a house light in the top back corner, which remained on during the behavioral procedures, and an infrared light in the top middle, which was not illuminated during this project. The left wall of the chamber consisted of a high-bright light, which was not illuminated during this project. All remaining slots of both walls were filled with blank metal panels. A USB camera was mounted on a steel panel outside the back Plexiglas wall of the chamber, angled at ~45°. The entire chamber was housed in an isolation cubicle with an acoustic liner and a house fan, which remained active during all behavioral procedures. Shock was delivered through the grid floor *via* a Precision Animal Shocker under the control of FreezeFrame 4 software, which also analyzed animal behavior in real-time. A freezing threshold of 2.0 was used as the scoring parameter for all animals. All video was recorded and stored for later analysis. The chamber walls were wiped with 70% isopropanol before use.

### Behavioral Procedures

Rats underwent contextual fear conditioning training and testing as described previously (Devulapalli et al., 2019, 2021; Orsi et al., 2019) in a Habitest chamber. Both experimental and naïve animals were handled for 4 days prior to behavioral training; the first two days occurred in the animal housing room and the second two days occurred in an adjacent room where behavioral training was to occur. Following this, the experimental group animals were placed into the fear conditioning apparatus and after a 1 min baseline, received 4 unsignaled footshock (1.0 mA, 1 sec) presentations. After a 1 min post-shock period, the animals were returned to their homecages. Naïve animals did not undergo the fear conditioning procedure and were not exposed to the conditioning chamber. We recently reported that male and female Sprague Dawley rats perform similarly on this task and do not differ in shock reactivity (Devulapalli et al., 2021), eliminating concerns of any biochemical effects seen between sexes being due to differences in behavioral performance or sensory processing.

### Tissue Collection

One or 4 h after the training session, experimental rats were overdosed on isoflurane in a necrosis chamber and the brain rapidly removed and immediately frozen on dry ice. During the same time on the training day, naïve animals were overdosed on isoflurane and the brain rapidly removed in the same manner as the experimental group. Tissue containing the basolateral amygdala (BLA) was then dissected out by blocking the brain in a rat brain matrix (Harvard Apparatus, Holliston, MA) incubated with dry ice. All dissected tissue was frozen at −80°C until needed.

### Tandem Ubiquitin Binding Entity (TUBE) Assays

BLA tissue was homogenized in buffer (10 mM HEPES, 1.5 mM MgCl_2_, 10 mM KCl, 0.5 mM DTT, 0.5% IGEPAL, 0.02% SDS, 70 mM NEM, 1 μl/ml protease inhibitor cocktail, and 1 μl/ml phosphatase inhibitor cocktail) designed to preserve endogenous polyubiquitinated chains. Then a high affinity K48 polyubiquitin-selective tandem ubiquitin binding entity (K48-TUBE, 100 μl, #UM407M, Life Sensors, Malvern, PA) conjugated to beads was washed in buffer (100 mM Tris-HCL, 150 mM NaCl, 5 mM EDTA, 0.08% NP-40) and a 500 μl mixture of protein (300 μg), protease inhibitor (1 μg/ml), and Wash Buffer was added, followed by incubation for 2 h on rotator at 4°C. Samples were then washed twice and incubated at 96°C for 5 min at 800 rpm in 1X sample buffer (Bio-rad, Hercules, CA). After cooling at room temperature, the supernatant was collected and stored at −80°C for mass spectrometry analysis.

### Liquid Chromatography Mass Spectrometry (LC/MS)

Optima™ LC/MS grade solvents, Pierce™ trypsin protease (MS grade), were from Fisher Scientific (Waltham, MA). S-Trap™ micro columns were from ProtiFi (Farmingdale, NY). Triethylammonium bicarbonate, pH 8.5 (TEAB), *o*-phosphoric acid, and formic acid were from MilliporeSigma (St. Louis, MO).

Protein samples were acidified by the addition of 11.1 μl 12% (v/v) *o*-phosphoric acid then protein precipitated by the addition of 725 μl LC/MS grade methanol and incubation at −80°C overnight. Precipitated protein was collected at the bottom of the sample tubes by centrifugation at room temperature for 15 mins at 13,000 x g. All but ~150 μl of the liquid from each sample was removed and discarded. Precipitated protein in each sample tube was then homogenized in the remaining liquid by scraping the sides of the tube with a pipette tip and repeated pipetting. The protein homogenate from each sample was then loaded onto an S-Trap™ micro column by centrifugation at room temperature for 1 min at 1000 x g. Each S-Trap™ micro column was washed four times with 150 μl LC/MS grade methanol at room temperature for 1 min at 1000 x g. Pierce™ trypsin protease (0.8 μg in 25 μl 50 mM TEAB) was loaded on top of each S-Trap™ micro column and incubated for 4 h at 37°C. A second aliquot of trypsin (0.8 μg in 25 μl 50 mM TEAB) was loaded on top of each S-Trap™ micro column and incubated overnight at 37°C.

Peptides were recovered by sequential washing of the spin column with 25 μl 50 mM TEAB, 25 μl solvent A (2:98 LC/MS grade acetonitrile: LC/MS grade water supplemented with 0.1% (v/v) formic acid), and 25 μl solvent B (80:20 LC-MS grade acetonitrile: LC-MS grade water supplemented with 0.1% (v/v) formic acid). Acetonitrile was removed using a centrifugal vacuum concentrator then peptide concentration was determined by measuring the absorbance at 215 nm using a DS-11 FX+ spectrophotometer/fluorometer (DeNovix, Wilmington, DE). Samples were diluted to 0.5 mg/ml using solvent A and 2 μl (1 μg, females) or 4 μl (2 μg, males) were analyzed using LC-MS/MS and each sample was analyzed twice yielding technical duplicates. The higher concentration used for males was due to a greater streptavidin signal than seen in females. As noted below, final values are normalized to streptavidin to account for this difference.

Samples were first loaded onto a precolumn (Acclaim PepMap 100 (Thermo Scientific, Waltham, MA), 100 μm x 2 cm) after which flow was diverted to an analytical column (50 cm μPAC (PharmaFluidics, Woburn, MA). The UPLC/autosampler utilized was an Easy-nLC 1,200 (Thermo Scientific, Waltham, MA). Flow rate was maintained at 150 nl/min and peptides were eluted utilizing a 2 to 45% gradient of solvent B in solvent A over 88 min. The mass spectrometer utilized was an Orbitrap Fusion Lumos Tribid™ from Thermo Scientific (Waltham, MA). Spray voltage on the μPAC compatible Easy-Spray emitter (PharmaFluidics, Woburn, MA) was 1,300 volts, the ion transfer tube was maintained at 275°C, the RF lens was set to 30% and the default charge state was set to 3.

MS data for the m/z range of 400–1,500 was collected using the orbitrap at 120,000 resolution in positive profile mode with an AGC target of 4.0e5 and a maximum injection time of 50 ms. Peaks were filtered for MS/MS analysis based on having isotopic peak distribution expected of a peptide with an intensity above 2.0e4 and a charge state of 2–5. Peaks were excluded dynamically for 15 s after 1 scan with the MS/MS set to be collected at 45% of a chromatographic peak width with an expected peak width (FWHM) of 15 s. MS/MS data starting at m/z of 150 was collected using the orbitrap at 15,000 resolution in positive centroid mode with an AGC target of 1.0e5 and a maximum injection time of 200 ms. Activation type was HCD stepped from 27 to 33.

Data were analyzed utilizing Proteome Discoverer 2.5 (Thermo Scientific, Waltham, MA) combining a Sequest HT and Mascot 2.7 (Matrix Science, Boston, MA) search into one result summary for each sample. Both searches utilized the UniProt reference *R. norvegicus* proteome database (downloaded July 28, 2020) and a common protein contaminant database provided with the Proteome Discoverer (PD) software package. Each search assumed trypsin-specific peptides with the possibility of 2 missed cleavages, a precursor mass tolerance of 10 ppm and a fragment mass tolerance of 0.1 Da. Sequest HT searches also included the PD software precursor detector node to identify MSMS spectra containing peaks from more than one precursor. Sequest HT searches included a fixed modification of carbamidomethyl at Cys and the variable modifications of oxidation at Met and loss of Met at the N-terminus of a protein (required for using the INFERYS rescoring node). Peptide matches identified by Sequest HT were subjected to INFERYS rescoring to further optimize the number of peptides identified with high confidence.

Mascot searches included the following dynamic modifications: oxidation of Met, acetylation of the protein N-terminus, cyclization of a peptide N-terminal Gln to pyro-Glu, N-ethylmaleimide at Cys, DeStreak (β-mercaptoethanol) at Cys, GlyGly at Lys, and deamidation of Asn/Gln residues.

Protein identifications were reported at a 1% false discovery rate (high confidence) or at a 5% false discovery rate (medium confidence) based on searches of decoy databases utilizing the same parameters as above. The software matched peptide peaks across all runs and protein quantities are the sum of all peptide intensities associated with the protein. Values were normalized to Streptavidin. Technical duplicates were averaged then biological replicates were averaged before determination of the trained to naïve ratio. Due to the loading differences between male and female samples (noted above), a simple *t*-test was used to determine *p*-values comparing the 5 trained males to the 5 naïve males and the 5 trained females to the 5 naïve females with p < 0.05 as threshold for a protein as being significantly different between group. All data and related files were submitted to the PRIDE archive with accession number PXD027544.

### Mass Spectrometry Pathway Analysis

All quantified ubiquitinated proteins were analyzed for identifying pathways and networks using Ingenuity Pathway Analysis (IPA) software (QIAGEN, Redwood City, CA) core analysis based on the user dataset as the reference set. The top canonical pathways and molecular networks associated with the uploaded dataset were listed along with the *p*-values calculated using a right tailed Fisher's exact test.

### Antibodies

Antibodies included synaptotagmin-1 (1:1000; #14558S Cell Signaling Technology, Danvers, MA), Cathepsin-D (1:1000, #69854, Cell Signaling Technology), Actin (1:1000; #4967, Cell Signaling Technology) and GFAP (1:5000; #ab190288, Abcam, Waltham, MA).

### Western Blot Analysis

Western blots were performed with 7% Acrylamide gels using 10 μg of protein as described previously (Orsi et al., 2019). Membranes were transferred with a Turbo Transfer System (Biorad) and incubated in a 50:50 blocking buffer (50% Licor TBS blocking buffer and 50% TBS + 0.1% Tween-20) for 1 h at room temperature, followed by overnight incubation in primary antibody in 50:50 blocking buffer at 4°C. Membranes were then washed 3 times for 10 min with TBS + 0.1% Tween-20 (TBSt) and incubated in secondary antibody (1:20,000; goat anti-rabbit or goat anti-mouse IgG1 800CW) in 50:50 blocking buffer for 45 min. After two 10 min washes in TBSt, the membranes were washed in TBS, imaged using the Odyssey Fc (LI-COR, Lincoln, NE) and visualized proteins were analyzed using Image Studio Ver 5.2. Samples were normalized to Actin.

### Statistical Analyses

All data are presented as mean with standard error, with scatter plots to identify individual samples (except in line graphs). Training data were analyzed with 2-way ANOVA and Fisher LSD *post-hoc* tests and western blot analysis was done with one-tailed *t*-test, which was based on the a priori prediction of the conformational experiment for the LC/MS data. Statistical outliers were defined as those samples that were two or more standard deviations from the mean and were determined by the outlier function in Prism.

## Results

### Identification of Sex-Specific K48 Polyubiquitin Protein Targets in the Amygdala After Fear Conditioning

**Figure 1:**
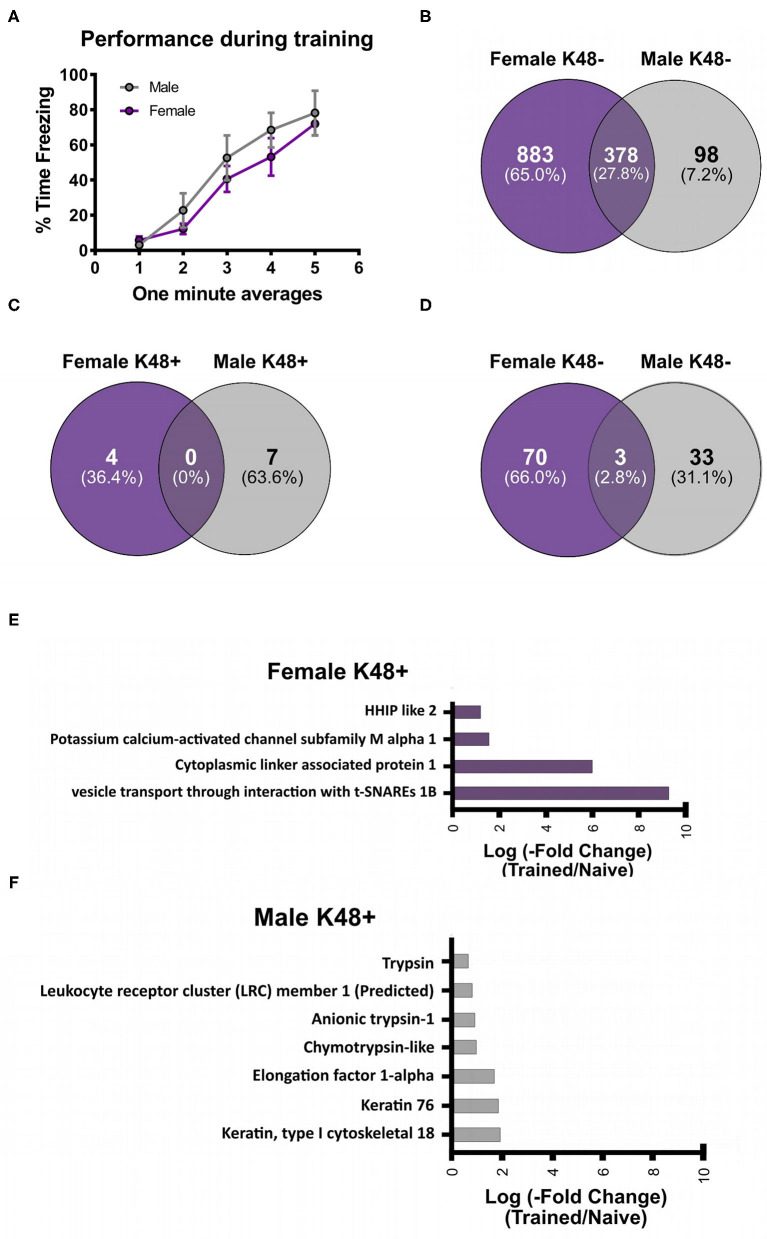
Sex-specific changes in K48 polyubiquitin protein targets in the amygdala of male and female rats during fear memory formation. Male and female Sprague-Dawley rats were trained to a foreground contextual fear conditioning task (*n* = 5 per sex) and the basolateral amygdala (BLA) was collected 1 h later. Samples were purified with a K48-specific tandem ubiquitin binding entity (K48-TUBE), target proteins identified with liquid chromatography mass spectrometry (MC/MS) and compared with naïve rats (*n* = 5 per sex). **(A)** Behavioral performance of animals during the training session. **(B)** Venn diagram showing the total K48 polyubiquitinated proteins identified in female (purple) and male (gray) BLA tissue. **(C,D)** Venn diagram showing the number of proteins that had increased **(C)** or decreased **(D)** abundance in BLA K48-purified samples 1 h after fear conditioning. **(E,F)** List of the proteins that had increased levels in K48-purified samples 1 h after training in females **(E)** and males **(F)** with their corresponding log value (-fold change).

To investigate the potential role of K48 polyubiquitination in fear memory formation, we trained male and female rats to contextual fear conditioning and collected the BLA 1 h later, which has consistently been reported as the peak time point for increased K48 polyubiquitination levels in the amygdala following fear conditioning (Jarome et al., 2011, 2013; Orsi et al., 2019; Devulapalli et al., 2021). Associative control animals were not used as we have consistently shown that exposing rats to the context and shock alone or in a non-associative manner (immediate shock training) do not engage protein degradation in the amygdala, as measured by changes in functional proteasome activity and K48 and proteasome-specific protein polyubiquitination (Jarome et al., 2011, 2013; Orsi et al., 2019). Proteins containing K48 polyubiquitination were then enriched using a K48-specific TUBE (Hjerpe et al., 2009) followed by protein identification using LC/MS (*N* = 5 per group per sex). This allowed an unbiased proteomic analysis of K48-specific polyubiquitin protein targets in the amygdala following fear conditioning. Behavioral performance of male and female rats during training is shown in Figure 1A. We found a main effect for Time [*F*_(4, 32)_ = 46.53, *P* < 0.0001], but not Sex [*F*_(1, 8)_ = 0.7709, *P* = 0.4055] and there was not a Time X Sex interaction [*F*_(4, 32)_ = 0.6453, *P* = 0.6342], indicating that male and female rats performed similar during the training session. Notably, while several labs have reported conflicting results in whether a sex difference exists on this task using the same species and strain as us (Graham et al., 2009; Russo and Parsons, 2021), we have previously reported that male and female rats show similar fear to the context during the testing session when using identical training parameters (Devulapalli et al., 2021). In total, we found 1,261 and 476 K48 polyubiquitinated proteins in females and males, respectively, of which 378 overlapped between sexes (Figure 1B). The larger amount of K48 polyubiquitin targets in the amygdala of females is consistent with our recent work showing that females have naturally higher levels of protein degradation in the amygdala in comparison to males (Devulapalli et al., 2021). Of these proteins, 77 in females and 43 in males had significantly altered expression levels of K48 polyubiquitination following fear conditioning based on the altered levels of protein identified in the samples (Supplementary Table 1), representing only a small fraction of the total K48 polyubiquitinated proteins in each dataset (Females = 6.1%; Males = 9.0%). Though these proteins varied across the subcellular compartments, a majority (> 50%) were localized to the cytoplasm in both sexes (Table 1).

**Table 1:** Subcellular localization of K48 polyubiquitin targets.

**Sex**	**Cytoplasm**	**Nucleus**	**Extracellular Space**	**Plasma Membrane**	**Other**	**Total**
**Male**	24	3	2	7	7	43
**Female**	41	15	4	11	6	77

Next, we separated these significantly altered K48 polyubiquitination protein targets by whether they had increased or decreased abundance in our TUBE purified samples following fear conditioning. The protein degradation process rapidly increases in the amygdala following fear conditioning, showing significant changes within 30 min and peaking at 1 h (Jarome et al., 2011). Since our proteomic analysis examined only the 1 h time point, we defined proteins with increased abundance in our enriched samples as targets that are about to be degraded while those with decreased abundance were defined as having actively undergone degradation; confirmation for this assumption will be discussed below (**Figure 6**). Importantly, we find that proteomic analyses on non-degradation polyubiquitin chains (M1) reveals few proteins with decreased abundance (Musaus et al., 2021), supporting that reduced levels in our K48 purified samples is likely indicative of the degradation process. Of the proteins with significantly altered levels, we identified 11 with increased abundance in K48-purified samples after fear learning, with 4 proteins found in females and 7 in males (Figure 1C), but no mutual proteins between the sexes. Conversely, both females and males had a higher number of proteins with significantly lower abundance in K48-purified samples following fear conditioning: 73 proteins of our dataset were identified in females and 36 proteins in males, with 3 of these proteins overlapping between sexes (Figure 1D). These observations suggest that there is a unique K48-mediated protein degradation pattern concerning protein targets between the sexes and that the learning-induced changes in the protein degradation process likely occur very rapidly following fear conditioning. All four proteins with increased abundance in K48-purified samples in females are shown in Figure 1E (*P* < 0.05), of which one protein, VTI1B, had a 593-fold increase compared to naïve controls. On the contrary, no proteins were identified to have a fold change higher than 4 in males (Figure 1F). Together, these results reveal that, contrary to our recent report which used broad immunoblotting approaches (Devulapalli et al., 2021), females do show significant upreguation of the protein degradation process in the amygdala during fear memory formation. Importantly, despite these mutual increases in protein degradation in the amygdala of males and females, the protein targets are largely unique between sexes.

### Sex Differences in the Functional Networks of K48 Polyubiquitin Protein Targets During Fear Memory Formation

**Figure 2:**
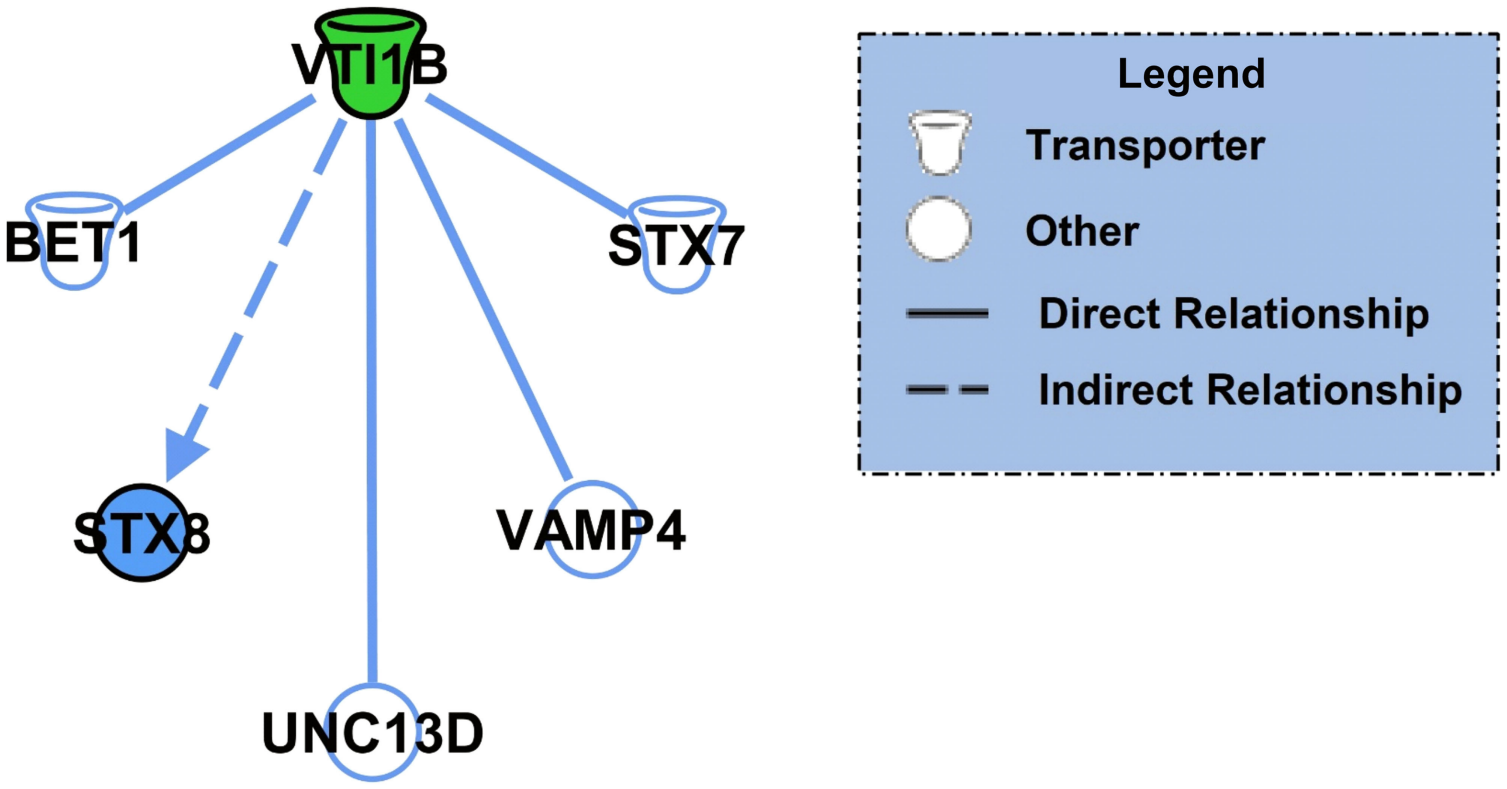
The downstream effectors of the putative K48 polyubiquitin target protein in the amygdala of females. Using IPA, the downstream proteins of VTI1B, which had the largest increase in abundance following K48 purification 1 h after the training in females, were identified. Green denotes a decrease in the protein expression levels, which leads to inhibition of the effector protein (shown in blue). Direct relationships are shown with solid lines and the indirect relationship is indicated by a dotted line.

After identifying the specific protein targets of K48 polyubiquitination in males and females, we first used IPA software to identify downstream targets of VTI1B, which is involved in vesicle transport pathways, an important process in synaptogenesis signaling pathways, to better interpret the dramatic increase in abundance observed in K48-purified samples from female BLA tissue. Since the increase in protein abundance following K48 purification indicates subsequent degradation of this substrate, we predicted the outcome of VTI1B loss on its downstream targets. Based on our analysis, 5 proteins interact with VTI1B or play a role in downstream pathway targets, of which syntaxin 8 (STX8) is positively regulated by VTI1B (Figure 2). Importantly, STX8 is mainly involved in protein trafficking *via* vesicle fusion and exocytosis by binding to proteins, such as VTI1B. These findings suggest a role for degradation in presynaptic plasticity in the female amygdala.

**Figure 3:**
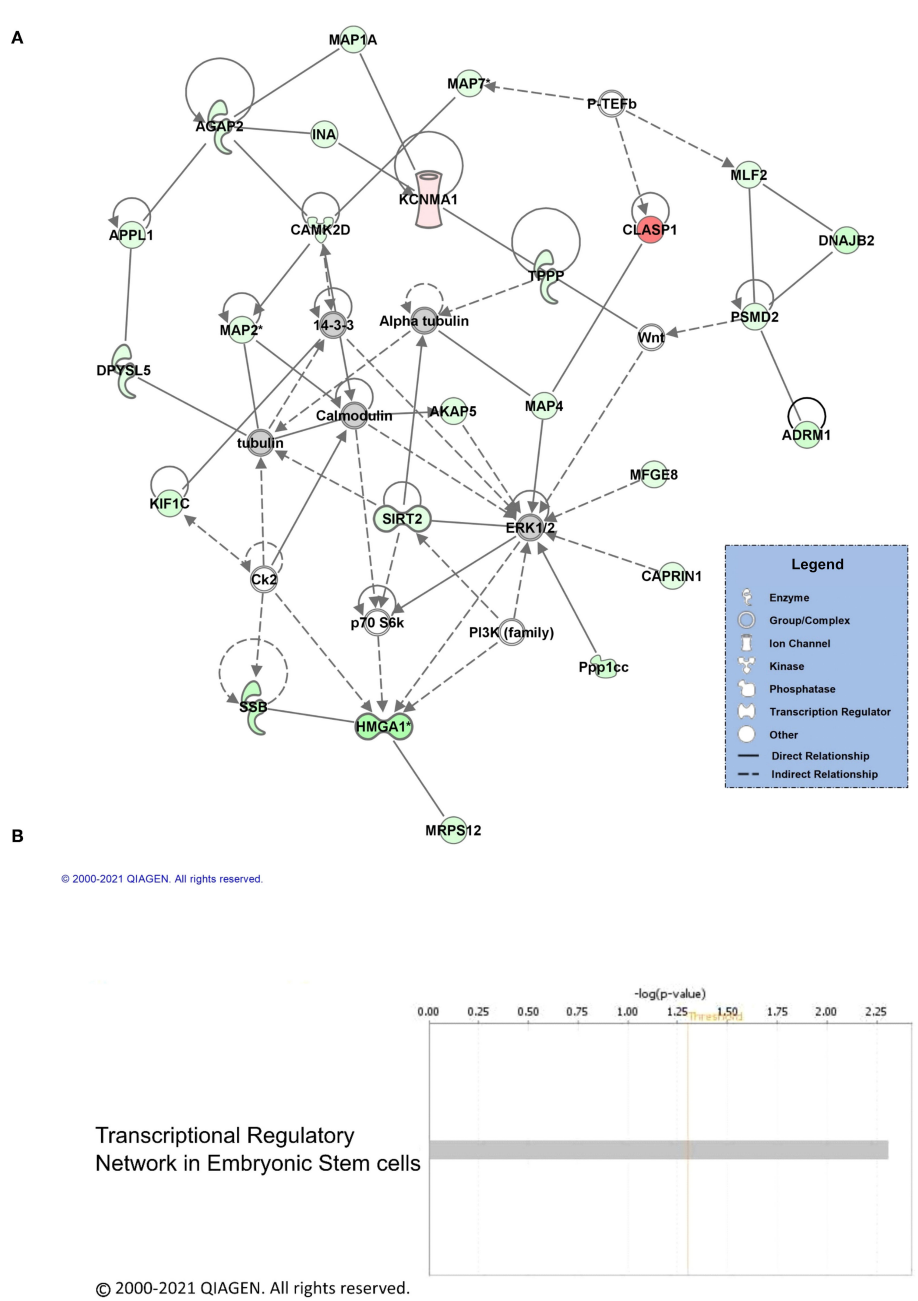
Pathway and network analysis of proteins regulated by K48 polyubiquitin in the amygdala of females. **(A)** Network of associated proteins with high fold change ratio of K48 polyubiquitin targeting in females was created using IPA. The proteins with lower levels in K48-purified samples 1 h after training are shown in green and those that had enhanced levels are shown in red. Greater fold-changes have brighter colors. Solid lines indicate a direct relationship, while dotted lines refer to indirect relationships between the proteins. **(B)** The canonical pathway associated with proteins differentially polyubiquitinated (K48) in the female amygdala detected by IPA.

Next, we performed core analysis on our male and female K48 datasets to determine whether any of the proteins that were identified as having significantly altered abundance in K48-purified samples interacted with each other in a network or a particular signaling pathway. We first examined the female dataset and IPA detected 5 different networks and one signaling pathway. The top network **(A**) and the canonical pathway (**B**) are shown in Figure 3. The total number of proteins in this network are 35, with the majority of them (23 proteins, indicated by green) showing decreased abundance, 2 showing an increase (red), and 10 (part of network but not identified by LC/MS) having no change in abundance following K48 purification. Additionally, many of these proteins are associated with microtubules and transportation within the cell, such as MAPs, TPPP, and KIF1C. Unlike the network of protein-protein interaction, only two proteins are contributing to the identified signaling pathway, transcriptional regulatory network in embryonic stem cells: H3.3 histone A (H3-3A/H3-3B) and SRY-box transcription factor 2 (SOX2). Both of these proteins have lower abundance in the K48-purified sample after fear conditioning.

**Figure 4:**
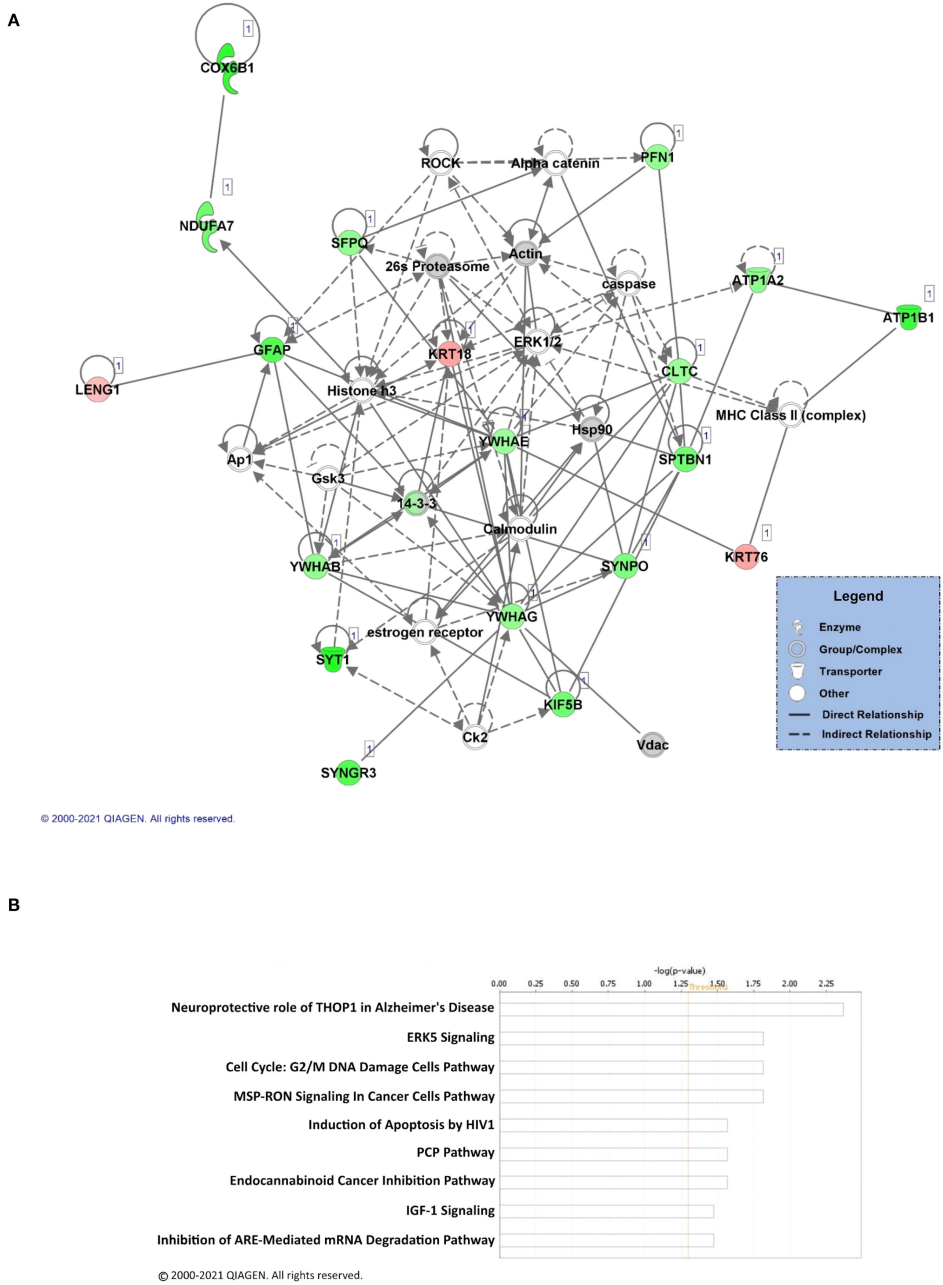
Pathway and network analysis of proteins regulated by K48 polyubiquitin in the amygdala of males. **(A)** Network of associated proteins with a high fold change ratio of K48 polyubiquitin targeting in males was created using IPA. The proteins with lower levels in K48-purified samples 1 h after training are shown in green and those that had enhanced levels are shown in red. Greater fold-changes have brighter colors. Solid lines indicate a direct relationship while dotted lines refer to indirect relationships between the proteins. **(B)** The top canonical pathways associated with the altered K48 polyubiquitin targets after the training in the male amygdala and their corresponding score (-log[p value]). A white bar indicates that there is no clear signal for activation or inhibition.

Next, we repeated the networks and pathways analyses for the male dataset. Similar to the female analysis, IPA detected 5 networks that contained interactions of the proteins identified as having significantly altered levels in K48-purified samples after fear learning. Moreover, the proteins with decreased abundance following K48 purification were more abundant in the top network (shown in green; Figure 4A). There are 35 proteins in this network in total, of which 17 had decreased, 3 had increased, and 15 (part of network but not identified by LC/MS) had no changes in abundance in K48-purified samples 1 h following training. Some of these identified proteins are associated with the cytoskeleton, ATP synthesis, and cell signaling (i.e., neurotransmitter transduction), such as the YWHA family, SPTBN1, and ATP1A2. However, proteins in the other 4 networks are also involved in some of the canonical pathways (Figure 4B); neuroprotective role of THOP1 in Alzheimer's disease, ERK5 signaling, and inhibition of ARE-mediated mRNA degradation pathway are among these pathways detected by IPA. Collectively, these results suggest that the protein targets of K48 polyubiquitination may have sex-specific functional roles during fear memory formation.

### Sex Independent K48 Polyubiquitin Targets During Fear Memory Formation

**Figure 5:**
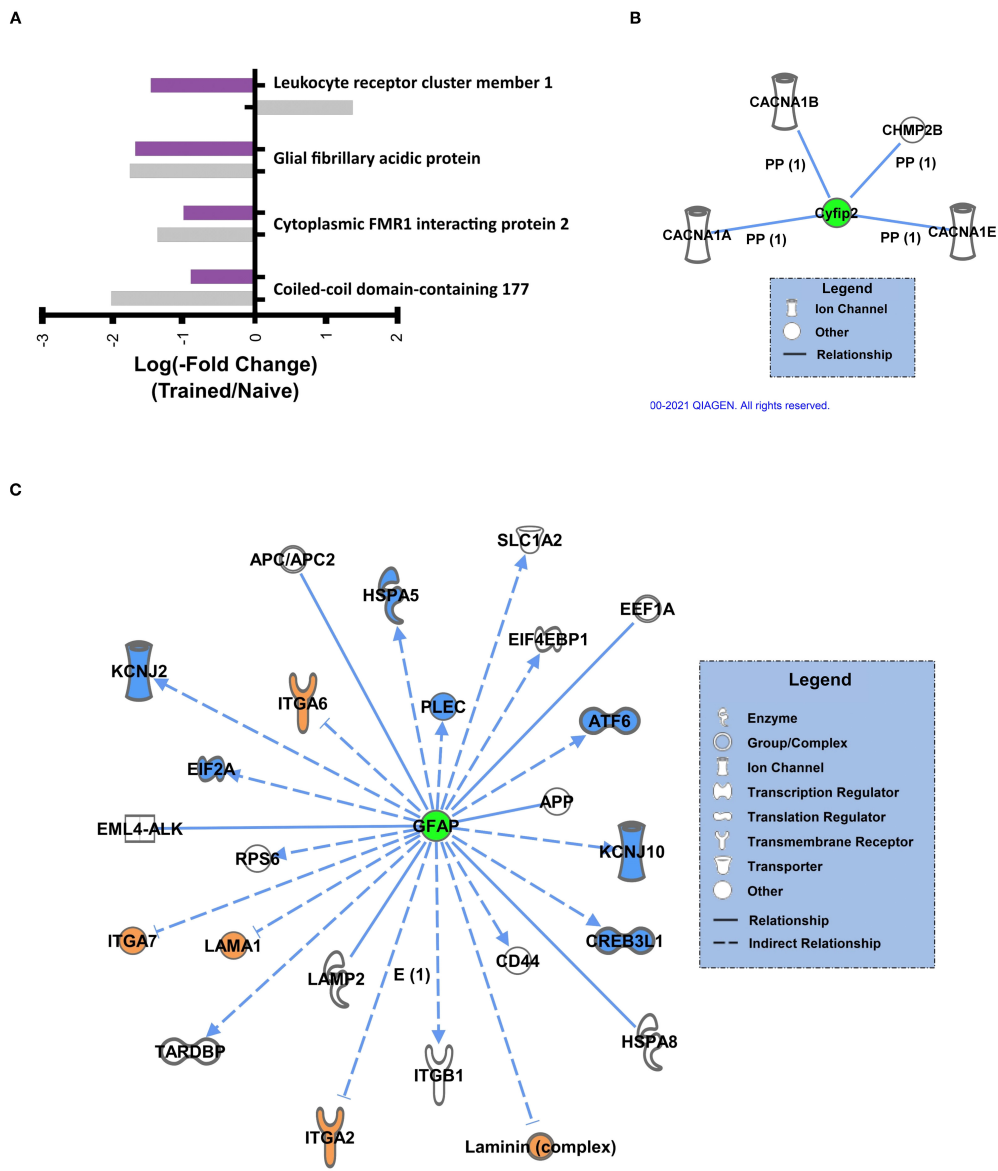
Sex-independent K48 polyubiquitin targets in the amygdala during fear memory formation **(A)** Proteins found to be significantly altered in both female and male K48-purified samples following fear conditioning, with corresponding log value (-fold change). **(B,C)** Using IPA, the downstream proteins of Cyfip2 **(B)** and GFAP **(C)** were identified. Green denotes a decrease in protein abundance in the K48-purified samples, which reflects reduced protein expression levels and leads to decreased (shown in blue) or increased (shown in orange) function/levels of the effector protein. Direct relationships are shown with solid lines and the indirect relationship is indicated by a dotted line.

We further examined the overlap in K48 polyubiquitin targets in the BLA of male and female animals. There were 3 proteins identified in both the male and female datasets as having significantly reduced levels in K48-purified samples following training (GFAP, Cyfip2, and CCDC177, Figure 5A). Of these, both GFAP and Cyfip2 are involved in cytoskeleton and cellular structural changes, but the function of CCDC177 is still unknown. Conversely, 1 protein, LENG1, was identified as having increased abundance in male K48-purified samples and decreased abundance in female K48-purified samples 1 h post-training, suggesting a more rapid degradation in the females, although the function of this protein is not well understood. Next, we looked at the downstream targets of these 4 proteins. IPA failed to detect any targets for CCDC177 and LENG1, which is consistent with the lack of available information regarding their function, but networks were generated for Cyfip2 (Figure 5B) and GFAP (Figure 5C). Reductions in the Cyfip2 protein do not change the activity or levels of its downstream target in rats. However, GFAP is a well known intermediate filament protein primarily expressed in astrocytes and interacts with many other proteins, mainly to maintain the structural integrity of astrocytes. Changes in this protein result in dramatic changes in a range of other proteins, such as transcription regulators, ion channels, and transmembrane receptors. Collectively, these findings suggest that distinct sets of proteins are targeted by K48-polyubiquitination after the fear learning to facilitate synaptic plasticity and morphological changes needed in females and males, except for a few proteins that their specific role is not well studied in memory and neuronal plasticity.

### K48 Polyubiquitin Protein Targets Have Reduced Expression Following Fear Conditioning

**Figure 6:**
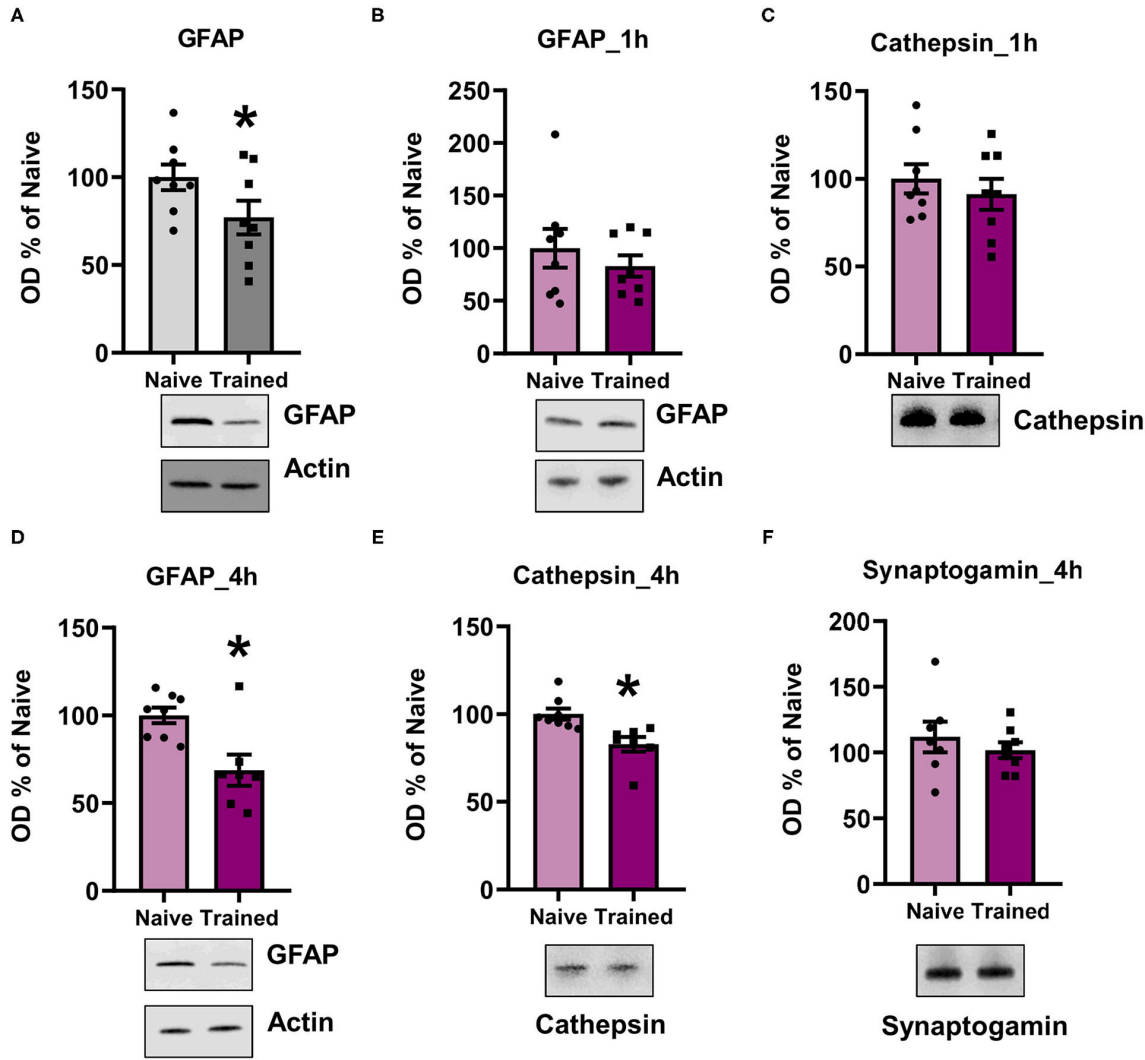
K48 polyubiquitination targets substrates for degradation in both males and females during fear memory formation. **(A,B)** In males **(A)** but not females **(B)**, GFAP levels were decreased in the basolateral amygdala (BLA) 1 h after contextual fear conditioning (*n* = 8 per group per sex). **(C)** Cathespin-D levels did not change in the BLA of females 1 h after contextual fear conditioning. **(D–F)** In females, GFAP **(D)** and Cathespin-D **(E)**, but not synaptogamin **(F)**, levels were decreased in the BLA 4 h after contextual fear conditioning (*n* = 7–8 per group). *P < 0.05 from Naïve.

To confirm the LC/MS results and that the K48 polyubiquitin mark was targeting substrates for degradation, we assessed the expression levels of candidate proteins with lower abundance in K48-TUBE enriched samples using western blotting. We first examined GFAP since it was the only major target that was altered in the same direction in both sexes (approximately -two-fold change in both; *N* = 5 per group per sex) and is a crucial component of astrocyte morphology. To increase power in this analysis, we included additional amygdala of naïve rats or those collected 1 h following contextual fear conditioning. In the amygdala of the males, there was a significant reduction in GFAP levels (t_14_ = 1.907, *p* =0.0387, N = 8 per group; Figure 6A). Conversely, females did not show any significant changes in GFAP levels (t_14_ = 0.804, *p* = 0.2174, *N* = 8 per group; Figure 6B). To confirm this, next, we quantified Cathepsin D (CTSD) levels, one of the proteins with a high fold-change reduction in female K48-TUBE enriched samples. Consistent with the GFAP result, there were no changes in the trained group CTSD levels compared to the naïve animals (t_14_ = 0.7264, *p* = 0.2398; Figure 6C). While unexpected, it is possible that increases in the degradation of a target substrate could take time to be reflected as a change in total protein level (Lee et al., 2008), especially considering that proteasome activity remains elevated for at least 4 h post-training (Jarome et al., 2013). Additionally, protein degradation is a multi-step process, which begins with deubiquitination of the protein before it enters the 19S cap of the proteasome, followed by degradation in the 20S core. Thus, it is possible that the proteins identified as having a lower level in the K48-TUBE enriched samples may have lost the K48 polyubquitination mark, but not yet been degraded, supporting the idea of active degradation. To confirm that the proteins identified in female K48-TUBE purified samples were undergoing degradation, we trained another group of females for contextual fear conditioning and collected their amygdala 4 h post-training (*N* = 7–8 per group). We found a significant reduction in both GFAP (t_13_ = 3.272, *p* = 0.0030; Figure 6D) and CTSD (t_13_ = 3.338, *p* = 0.0027; Figure 6E) levels, but not synaptogamin (t_13_ = 0.8033, *p* = 0.2181; Figure 6F), which was not indicated as a target in females in our proteomic analysis, in the trained animals compared to the naïve controls. These results confirm that the decreased fold-changes in TUBE enriched samples were due to K48 polyubiquitin targeting substrates for degradation by the proteasome, which is consistent with its canonical function.

## Discussion

Although the ubiquitin-proteasome system has been consistently implicated as a critical regulator of fear memory formation in several brain regions, including the amygdala, the specific proteins being targeted by the protein degradation process have yet to be elucidated (Lopez-Salon et al., 2001; Jarome et al., 2011; Rosenberg et al., 2016; Cullen et al., 2017; Orsi et al., 2019; Dulka et al., 2020; Devulapalli et al., 2021). Additionally, our group has recently demonstrated that there is a sex-specific role for ubiquitin-proteasome signaling in fear memory formation in the amygdala (Devulapalli et al., 2021), though little is known about the functional significance of protein degradation to fear memory consolidation and how this varies between males and females. Here, we performed the first unbiased proteomic analysis of K48 polyubiquitin protein targets in the amygdala of male and female rats following fear conditioning. To the best of our knowledge, this study provides the first proteome-wide insight into protein targets of degradation-specific K48 polyubiquitination during fear memory formation in both male and female rodents. Importantly, we provide strong evidence that sex differences exist in the protein targets of the degradation process in the amygdala following fear conditioning. Additionally, although in males and females the major cellular pathways targeted by K48 polyubiquitination overlapped in some cases, the specific proteins did not. Together, these results provide critical information that fills the gap in knowledge concerning the proteins being targeted by protein degradation during fear memory formation in both male and female rodents.

In this study, LC/MS was conducted to identify and quantify levels of individual proteins that had increased or decreased abundance in K48-purified amygdala samples following fear conditioning. This altered abundance indicates changes in K48 targeting of the specific proteins following fear conditioning, which we defined reduced abundance as indication of active degradation of the substrate. However, it should be noted that another interpretation could exist as it is possible that the target protein could have lost the K48 polyubiquitin mark through deubiquitination, without undergoing degradation by the proteasome. Our western blot data suggests that this is unlikely, though, as we confirmed that several proteins identified as targets of K48 polyubiquitin in our LC/MS analysis had reduced expression following fear conditioning. These data indicate that K48 polyubiquitination was likely targeting these substrates for degradation during the memory consolidation process, consistent with its canonical function in the UPS. Interestingly, one of the main targets that overlapped in K48 targeting in both sexes was GFAP, a critical component regulating astrocyte morphology. Specifically, GFAP has known roles as an intermediate filament for structural support of mature astrocytes (Eng et al., 2000) and studies suggest that astrocytes undergo morphological changes following activity (Choi et al., 2016). Therefore, K48 polyubiquitination-mediated degradation of GFAP after fear conditioning may be critically involved in astrocyte plasticity that occurs during memory formation, though the cell-type specific role of protein degradation to memory formation has never been directly tested.

The proteomic analysis revealed 11 proteins with significantly enhanced levels following K48 purification in the amygdala of males and females, with no overlap in protein targets between sexes. Conversely, 106 proteins had decreased abundance in K48-purified samples collected from the amygdala following fear conditioning, of which only 3 proteins overlapped between sexes. This minimal overlap supports an important sex-specific role for protein degradation in fear memory formation. In our previous study, we reported that protein degradation *via* K48 polyubiquitination was not increased in the amygdala of females following learning, though it was increased in males (Devulapalli et al., 2021). These results were obtained through western blot analysis, which, unlike LC/MS, quantified the global level of K48 polyubiquitination in the nuclear fraction and was not specific or sensitive enough to identify the changes in K48 polyubiquitin levels of individual proteins in the female amygdala. Importantly, the larger deficit between the number of proteins with increased as opposed to decreased levels following K48 purification in females and the minimal overlap in protein targets between sexes may explain why our previously reported western blot analysis was unable to detect changes in K48 polyubiquitination following fear conditioning in the female amygdala. Based on these findings, it is clear that females do engage protein degradation in the amygdala during fear memory formation. The current findings provide a greater context for understanding sex-differences in the role of degradation-specific K48 polyubiquitination during fear memory formation in the amygdala.

To better understand the role of the proteins being targeted by K48 polyubiquitination, pathway analysis was conducted. The most notable pathways identified in males were the neuroprotective role of THOP1, DNA damage pathways, and ERK5 signaling, all of which have been previously implicated in memory formation. For example, THOP1 knockout mice have impaired memory retention (Santos et al., 2019), and our group and others have previously reported that DNA double-stranded breaks occur and are necessary for memory formation and modification in the hippocampus (Madabhushi et al., 2015; Navabpour et al., 2020), though this has yet to be reported in the amygdala. Furthermore, ERK/MAPK signaling has long been implicated in memory consolidation in the amygdala and other brain regions (Chwang et al., 2006; Ehrlich and Josselyn, 2016). Collectively, the pathways identified in our study as targets of learning-related changes in protein degradation in the male amygdala support previous reports of specific molecular processes and signaling pathways involved in fear memory formation. Alternatively, in females, transcriptional regulatory network in embryonic stem cells was the only significant pathway identified. Although the pathway is broad, several important genes related to embryonic stem cells have also been implicated in memory formation, such as WNT and SHH (Maguschak and Ressler, 2011; Hung et al., 2014), and other factors, such as SOX2, have been implicated in glia and neuron functioning (Mercurio et al., 2019). Importantly, though the protein targets and pathways identified in males and females contain little overlap, there was likely a shared role for structural remodeling, which is based on the many overlapping non-ubiquitinated proteins related to the cytoskeleton that were identified in our network analyses between sexes. Collectively, these data suggest that while the protein targets of K48 polyubiquitination appear to be largely different between males and females, there may be a shared role for structural remodeling between sexes, though more research will be needed to confirm this observation.

The findings presented here provide novel insight into the specific proteins that are being targeted by K48 polyubiquitination in the amygdala of male and female rats following fear conditioning. However, there is still much to be discovered about the specific role of K48 polyubiquitination-related signaling during fear memory formation. One limitation of our study was that we did not directly manipulate K48 polyubiquitination and test the effects on memory and target substrate stability. Since K48 is a linkage site on the ubiquitin protein, which has multiple (4) coding genes, it is difficult to specifically manipulate endogenous K48 polyubiquitination without altering other lysine-linkage sites. In fact, due to these difficulties, to date, there is only indirect evidence for degradation-specific K48 polyubiquitination in fear memory formation, which comes from proteasome manipulations (Lopez-Salon et al., 2001; Artinian et al., 2008; Lee et al., 2008; Jarome et al., 2011; Rodriguez-Ortiz et al., 2011; Reis et al., 2013; Felsenberg et al., 2014; Figueiredo et al., 2015; Furini et al., 2015; Rosenberg et al., 2016; Devulapalli et al., 2021). However, such manipulations are not specific to K48 and actually cause an artificial accumulation of many forms of polyubiquitinated proteins, which can deplete the free ubiquitin pool. As a result, while the evidence obtained through proteasome manipulation is valuable, other lysine-linkage sites besides K48 that do and do not act as a degradation mark would be affected by the manipulations of the proteasome, making interpretation of results difficult. This may explain why SHANK, MOV10 and IkB were not identified as significant targets of K48 in either sex during fear memory formation in our dataset, despite previous evidence demonstrating proteasome-mediated degradation of these proteins following learning (Lopez-Salon et al., 2001; Yeh et al., 2002; Lee et al., 2008; Jarome et al., 2011). Furthermore, this could have occurred due to these putative degradation targets being marked by a polyubiquitin chain carrying another linkage site, such as K11 (Xu et al., 2009; Meyer and Rape, 2014; Meza Gutierrez et al., 2018; Musaus et al., 2020). Unfortunately, until more precise technology is developed, the specific role of K48 polyubiquitination in fear memory formation in any brain region will remain equivocal. Identifying a method for specifically manipulating lysine-specific ubiquitin linkage sites will be key for elucidating the roles of K48 polyubiquitination during fear memory formation, as well as whether other ubiquitin linkage sites have similar roles and targets between the sexes following fear conditioning.

Another limitation could be the time of tissue collection. In the present study, amygdala tissue was only collected 1 h after fear conditioning, which has been consistently reported by our group as the earliest peak in protein degradation after training (Jarome et al., 2011, 2013; Reis et al., 2013; Orsi et al., 2019; Devulapalli et al., 2021). However, later time points, such as 4 h, maintain significant levels of protein degradation in the amygdala (Jarome et al., 2013) and other brain regions (Lopez-Salon et al., 2001; Rosenberg et al., 2016). Additionally, protein homeostasis is necessary as protein synthesis and degradation are both required for long-term memory (Fonseca et al., 2006; Cajigas et al., 2010). Therefore, it is possible then that unique proteins may be targeted by K48 polyubiquitination at different time points following fear conditioning, as protein degradation remains at a significant level for several hours. Thus, identifying targets of K48 polyubiquitination at different time points after fear conditioning may identify new proteins involved in memory consolidation and help elucidate the potential roles of K48 polyubiquitination in this process. Despite this, the findings presented here still provide critical insight into the sex-specific K48 polyubiquitin protein targets in the amygdala during fear memory formation. Future studies should use this K48-TUBE-LC/MS approach to examine how the protein degradation targets change across time during the memory consolidation process.

In the present study associative control animals were not used due to our previous reports which demonstrate that exposing rats to the context and shock alone or in a non-associative manner (immediate shock training) does not engage protein degradation in the amygdala, as measured by changes in functional proteasome activity and K48 and proteasome-specific protein polyubiquitination (Jarome et al., 2011, 2013; Orsi et al., 2019). However, these previous studies were only conducted using male rodents, so it is unclear if shock or contextual stimuli alone would induce protein degradation changes in the amygdala of female rats. Therefore, it is possible that some of the K48 polyubiquitin protein targets identified in the amygdala of females could be engaged as a result of context or shock alone, independent of associative learning. While in females we cannot completely dissociate those proteins that are targeted by K48 polyubiquitination in response to the context-shock association compared to either stimuli independently, our findings still indicate an important sex effect in the proteins being targeted for protein degradation within the amygdala following fear conditioning. Further validation of the learning-specificity of these K48 polyubiquitin targets in females will need to be addressed in future studies.

In conclusion, we present the first detailed analysis of sex differences in the target substrates of the proteasome-mediated protein degradation process in the amygdala during fear memory formation. These results provide the first evidence suggesting potential sex-specific functions of the K48 polyubiquitination-mediated protein degradation process during fear memory formation, which could have important implications for understanding the etiology of sex-related differences in the formation of fear memories that may underlie post-traumatic stress disorder. However, it should be noted that as we were unable to confirm that the animals used in the present study presented behaviors characteristic of PTSD, we cannot conclude that these identified targets of protein degradation are pathological in nature. Instead, our findings may have more direct implications in sex-dependent protein degradation processes underlying learning and memory in general, which could significantly advance our understanding of how ubiquitin proteasome activity contributes to memory formation in cells.

## Data Availability Statement

The original contributions presented in the study are publicly available. This data can be found here: https://www.ebi.ac.uk/pride/archive/, PXD027544.

## Ethics Statement

The animal study was reviewed and approved by Virginia Polytechnic Institute and State University Institutional Animal Care and Use Committee (protocol #18-019).

## Author Contributions

KF, MM, and TJ designed the experiments. KF, MM, and KM conducted the experiments. WR and RH performed mass spectrometry. SN performed analysis of mass spectrometry data. KF, SN, MM, and TJ wrote the manuscript. All authors edited, reviewed, and approved the manuscript for publication.

## Funding

This work was supported by National Institute of Health (NIH) Grants MH120498, MH120569, MH122414, and MH123742 (TJ). The funder had no role in the project development, data interpretation or decision to publish.

## Conflict of Interest

The authors declare that the research was conducted in the absence of any commercial or financial relationships that could be construed as a potential conflict of interest.

## Publisher's Note

All claims expressed in this article are solely those of the authors and do not necessarily represent those of their affiliated organizations, or those of the publisher, the editors and the reviewers. Any product that may be evaluated in this article, or claim that may be made by its manufacturer, is not guaranteed or endorsed by the publisher.

## Abbreviations

ATP1A2: ATPase Na+/K+ Transporting Subunit Alpha 2
CCDC177: Coiled-Coil Domain Containing 177
CTSD: Cathepsin D
Cyfip2: Cytoplasmic FMR1 Interacting Protein 2
ERK5: Extracellular-Signal-Regulated Kinase 5
GFAP: Glial Fibrillary Acidic Protein
GKAP: guanylate kinase-associated protein
IκB: Inhibitory kappa B
KIF1C: Kinesin Family Member 1C
LENG1: Leukocyte Receptor Cluster Member 1
MAP: mitogen-activated protein
MOV10: Moloney leukemia virus 10
SHANK: SH3 and multiple ankyrin repeat domains 3
SHH: Sonic hedgehog
SOX2: SRY-box transcription factor 2
SPTBN1: Spectrin Beta, Non-Erythrocytic 1
STX8: syntaxin 8
THOP1: Thimet Oligopeptidase 1
TPPP: Tubulin polymerization-promoting protein
VTI1B: vesicle transport through interaction with t-SNAREs 1B
WNT: Wingless/Integrated
YWHA: 14-3-3 phospho-serine/phosphor-threonine binding proteins.
