# Supplementary material for: Proteomic Analysis Reveals Sex-Specific Protein Degradation Targets in the Amygdala During Fear Memory Formation
Source: Front Mol Neurosci. 2021 Sep 29;14:716284. doi: 10.3389/fnmol.2021.716284 (PMC8511838; doi:10.3389/fnmol.2021.716284)
Supplement: Supplementary file 1 [file Table_1.DOCX]

**Supplemental Table 1: K48 polyubiquitin targets in the amygdala of male and female rats**

| **Sex** | **Protein** | **Log ratio** | ***p* value** |
| --- | --- | --- | --- |
| Female | ATP5IF1 | -3.373 | 5.12E-05 |
| Female | DNAJB2 | -2.409 | 0.000293 |
| Female | FGF12 | -2.124 | 0.000318 |
| Female | CNKSR1 | -3.523 | 0.000615 |
| Female | TPRG1L | -2.493 | 0.0013 |
| Female | HMGA1 | -3.87 | 0.00157 |
| Female | PDF | -2.224 | 0.00179 |
| Female | DACT3 | -2.061 | 0.00254 |
| Female | KIF1C | -2.105 | 0.00336 |
| Female | RBM14 | -2.06 | 0.00367 |
| Female | CCT4 | -2.391 | 0.00519 |
| Female | MAP7 | -1.171 | 0.00531 |
| Female | MAP2 | -0.567 | 0.00543 |
| Female | SOX2 | -1.656 | 0.00559 |
| Female | IDH1 | -1.55 | 0.00591 |
| Female | TPPP | -0.936 | 0.00596 |
| Female | Uba52 | -1.804 | 0.00643 |
| Female | PSMD2 | -1.004 | 0.00684 |
| Female | HMGN2 | -3.121 | 0.00727 |
| Female | CRACDL | -1.135 | 0.00778 |
| Female | MLF2 | -0.764 | 0.00855 |
| Female | CAPRIN1 | -1.351 | 0.00891 |
| Female | MAP1A | -0.639 | 0.0104 |
| Female | Gar1 | -1.02 | 0.0124 |
| Female | CDKL5 | -1.598 | 0.0131 |
| Female | APPL1 | -1.622 | 0.0134 |
| Female | RBM3 | -1.135 | 0.0134 |
| Female | SH3BGRL3 | -2.327 | 0.0136 |
| Female | ADRM1 | -2.35 | 0.0138 |
| Female | Btbd8 | -1.613 | 0.0148 |
| Female | Ppp1cc | -2.272 | 0.015 |
| Female | RPS26 | -1.174 | 0.0156 |
| Female | LENG1 | -1.517 | 0.0159 |
| Female | CCDC177 | -0.83 | 0.0171 |
| Female | PRRC2A | -1.021 | 0.0172 |
| Female | CDS2 | -2.136 | 0.0177 |
| Female | KCNMA1 | 1.443 | 0.0178 |
| Female | AKAP5 | -1.639 | 0.0191 |
| Female | FBLIM1 | -2.197 | 0.0202 |
| Female | TKFC | -7.07 | 0.0215 |
| Female | AGAP2 | -1.698 | 0.0223 |
| Female | TMA7 | -1.201 | 0.0229 |
| Female | CSN2 | -1.638 | 0.0253 |
| Female | GFAP | -1.558 | 0.0256 |
| Female | HP1BP3 | -0.836 | 0.0265 |
| Female | MYH10 | -0.824 | 0.0266 |
| Female | TAGLN3 | -1.717 | 0.0276 |
| Female | ARF5 | -2.873 | 0.0281 |
| Female | SSB | -2.859 | 0.0296 |
| Female | EEF1A2 | -0.446 | 0.0308 |
| Female | DPYSL5 | -1.008 | 0.0309 |
| Female | SIRT2 | -1.527 | 0.031 |
| Female | CAMK2D | -1.218 | 0.0315 |
| Female | MRPL34 | -1.031 | 0.0315 |
| Female | C5orf24 | -0.971 | 0.0321 |
| Female | HHIPL2 | 1.152 | 0.0328 |
| Female | CTSD | -3.709 | 0.0331 |
| Female | PDIA3 | -0.992 | 0.0331 |
| Female | EEF1D | -1.481 | 0.0354 |
| Female | ACTR3B | -2.733 | 0.036 |
| Female | MFGE8 | -0.895 | 0.0367 |
| Female | H3-3A/H3-3B | -0.875 | 0.0373 |
| Female | Pcmt1 | -1.724 | 0.038 |
| Female | MAP4 | -0.653 | 0.0383 |
| Female | MRPS12 | -2.022 | 0.0387 |
| Female | Cyfip2 | -0.99 | 0.0408 |
| Female | PHPT1 | -1.043 | 0.0408 |
| Female | 4930544G11Rik | -1.377 | 0.0412 |
| Female | SLC30A3 | -2.711 | 0.042 |
| Female | CLASP1 | 6 | 0.0421 |
| Female | INA | -1.27 | 0.0432 |
| Female | NDUFA9 | -1.549 | 0.0456 |
| Female | MFSD6 | -1.907 | 0.0465 |
| Female | VTI1B | 9.212 | 0.0477 |
| Female | LOC100360846/Psmb6 | -1.202 | 0.0489 |
| Female | PLXNA1 | -1.039 | 0.0491 |
| Female | CAPZA2 | -1.168 | 0.0496 |
| Male | KRT76 | 1.862 | 0.00064 |
| Male | KRT18 | 1.914 | 0.00286 |
| Male | G3bp2 | -0.57 | 0.00316 |
| Male | LOC100360413/LOC100911991 | 1.849 | 0.00398 |
| Male | PRSS2 | 1.517 | 0.00481 |
| Male | PFN1 | -1.101 | 0.00687 |
| Male | ATP1B1 | -1.946 | 0.00698 |
| Male | SYNGR3 | -1.711 | 0.00787 |
| Male | LOC100911847 | -1.041 | 0.00795 |
| Male | YWHAB | -1.134 | 0.00824 |
| Male | KCNAB2 | -1.118 | 0.00879 |
| Male | Cyfip2 | -1.297 | 0.00911 |
| Male | SFPQ | -1.125 | 0.0101 |
| Male | CLTC | -1.037 | 0.0131 |
| Male | KIF5B | -1.437 | 0.0135 |
| Male | ZC2HC1A | -0.719 | 0.0146 |
| Male | UBE2M | -0.917 | 0.015 |
| Male | GNAO1 | -1.6 | 0.0164 |
| Male | Trypsin | 1.25 | 0.0166 |
| Male | MRPL14 | -1.051 | 0.0175 |
| Male | SERINC1 | -0.607 | 0.0175 |
| Male | RHOA | -1.227 | 0.0177 |
| Male | CKB | -1.516 | 0.0178 |
| Male | HK1 | -1.04 | 0.0184 |
| Male | GLUL | -0.953 | 0.0191 |
| Male | CCDC177 | -2.023 | 0.0193 |
| Male | H2AZ2 | -1.669 | 0.0195 |
| Male | COX6B1 | -2.04 | 0.0207 |
| Male | CTRL | 1.553 | 0.0218 |
| Male | SYNPO | -1.288 | 0.0234 |
| Male | Hnrnpa3 | -0.747 | 0.0248 |
| Male | YWHAE | -1.02 | 0.0269 |
| Male | CAMK2B | -0.777 | 0.0301 |
| Male | LENG1 | 1.421 | 0.0302 |
| Male | YWHAG | -1.13 | 0.0311 |
| Male | ATP1A2 | -1.157 | 0.0325 |
| Male | SLC25A4 | -1.871 | 0.0366 |

| Male | SLC25A3 | -1.261 | 0.0369 |
| --- | --- | --- | --- |
| Male | NDUFA7 | -1.447 | 0.0411 |
| Male | GFAP | -1.711 | 0.043 |
| Male | PUSL1 | -1.367 | 0.0446 |
| Male | HBB | -1.451 | 0.0459 |
| Male | SYT1 | -2.211 | 0.0468 |
|  |  |  |  |
